# Community perceptions of a biopsychosocial model of integrated care in the health center: the case of 4 health districts in South Kivu, Democratic Republic of Congo

**DOI:** 10.1186/s12913-023-10455-1

**Published:** 2023-12-18

**Authors:** Bertin Kasongo, Abdon Mukalay, Christian Molima, Samuel Lwamushi Makali, Christian Chiribagula, Gérard Mparanyi, Hermès Karemere, Ghislain Bisimwa, Jean Macq

**Affiliations:** 1 Ecole Régionale de Santé Publique (ERSP), Catholic University of Bukavu, Bukavu, Democratic Republic of Congo; 2 School of Public Health (ESP), University of Lubumbashi, Lubumbashi, Democratic Republic of Congo; 3 Faculty of Pharmaceutical Sciences and Public Health, Official University of Bukavu, Bukavu, Democratic Republic of Congo; 4 Institute of Health and Society (IRSS), Université Catholique de Louvain, Brussels, Belgium

**Keywords:** Health center, Biopsychosocial, Integration of care, Perceptions, Community participation, Person-centered health care

## Abstract

**Background:**

Biopsychosocial care is one of the approaches recommended in the health system by the WHO. Although efforts are being made on the provider side to implement it and integrate it into the health system, the community dynamic also remains to be taken into account for its support. The objective of this study is to understand the community's perceptions of the concept of integrated health care management according to the biopsychosocial approach (BPS) at the Health Center of a Health District and its evaluation in its implementation.

**Methods:**

This cross-sectional study was done in six Health Areas belonging to four Health Districts in South Kivu, DRC. We conducted 15 semi-directive individual interviews with 9 respondents selected by convenience, including 6 members of the Development Committees of the Health Areas, with whom we conducted 12 interviews and 3 patients met in the health centers. The adapted Normalization MeAsure Development (NoMAD) tool, derived from the Theory of the Normalization Process of Complex Interventions, allowed us to collect data from November 2017 to February 2018, and then from November 2018 to February 2019. After data extraction and synthesis, we conducted a thematic analysis using the NoMAD tool to build a thematic framework. Six themes were grouped into three categories.

**Results:**

Initially, community reports that the BPS approach of integrated care in the Health Centre is understood differently by providers; but then, through collective coordination and integrated leadership within the health care team, the approach becomes clearer. The community encouraged some practices identified as catalysts to help the approach, notably the development of financial autonomy and mutual support, to the detriment of those identified as barriers. According to the community, the BPS model has further strengthened the performance of health workers and should be expanded and sustained.

**Conclusions:**

The results of our study show the importance of community dynamics in the care of biopsychosocial situations by providers. The barriers and catalysts to the mechanism, both community-based and professional, identified in our study should be considered in the process of integrating the biopsychosocial model of person-centered health care.

**Supplementary Information:**

The online version contains supplementary material available at 10.1186/s12913-023-10455-1.

## Background

The concept of person-centered care is one of the approaches increasingly promoted in the health system, in the sense of considering a person not only from a somatic point of view, but in his or her entirety by offering holistic care, i.e. physical, mental, emotional and socio-spiritual care [1–4]. This means taking into account the patient's needs, preferences, opinions and values. It also means taking care of the individual according to biopsychosocial considerations while also strengthening solidarity and community engagement [5–7].

To speak of integration of this approach in the care system, particularly at the front line of care, means that the health care team provides quality and safe care in a sustainable way, in its totality, around the health issues of each individual sufferer, offering preventive, curative, promotive, palliative and rehabilitative services. The care provided is juxtaposed with the specific needs of the person beyond the disease itself, hence, some level of collective coordination would be required to address complex and chronic problems. In this case, integrated care responds to the person-centered care approach [8–11].

Several studies have tried to analyze this biopsychosocial approach and its integration in specific situations such as in the field of mental health [4, 12–14]. Others have also developed integrative models for the holistic management of diseases and their consequences, including HIV [15] and diabetes [16] with an emphasis on collective coordination.

Confronted with armed conflict and sexual violence for the past twenty years, especially in the east, the Democratic Republic of Congo (DRC) has experimented with a holistic approach via several organizations to support the care of victims of sexual violence while integrating the biopsychosocial components [17, 18].

This care is offered for this type of situation (chronic pathologies, mental illness) by limiting itself to the care-giver relationship, without going beyond the individual dimension. Apart from this practice of care provision in which people are not seen in an integrated way [5], little data is available on the place of the community in the implementation of the biopsychosocial approach, its role and responsibility and its involvement. Yet, the recognition and documentation of community work remains of great importance in the pursuit of health sector reform [3, 19].

In order to improve the quality of care offered at the level of first-level care structures, a research and development project was implemented in South Kivu in the DRC, financed by Belgian cooperation (ARES-CCD), with the objective of reorganizing care at the level of health centers according to a new person-centered care approach [6].

As an initial analysis for the implementation of the approach, a survey was conducted among first-level health staff to identify barriers and facilitators to the approach [6]. Based on the opinions of the health workers, barriers and facilitators were identified to the shift from a disease control approach to a biopsychosocial approach.

In this article, as a complement to that study, we will present the community’s perceptions on the understanding and acceptance of the biopsychosocial approach by health workers, as well as the place of community leaders in the implementation of this approach. The level of knowledge and understanding of a concept in an environment is one of the factors that promote its acceptance and integration [20–23].

In order to allow effective integration of intervention in the health sector, it would be necessary to take into account both changes in the delivery of health care and community engagement to influence this mode of delivery as well [3]. This means that the community should be involved as a stakeholder and beneficiary of this intervention and directly involved in the implementation of any proposed changes in the organization of care.

Therefore, our study aims to understand the community's perceptions on the concept of integrated health care management according to the biopsychosocial approach (BPS) at the Health Center of a Health District and its evaluation in its implementation.

## Methods

### Study settings

This study was conducted in six health areas (HAs) in four Health Districts (HDs) in the province of South Kivu, DRC. These HDs are where the Research and Development Project (RDP), on which this study is based, is being implemented. They are one urban health district (Bagira Health District) and three rural health districts (Katana Health District, Miti-Murhesa Health District and Walungu Health District). The health areas concerned by the study are those of Lumu and Nyamuhinga in Bagira, Kabushwa in Katana, Lwiro in Miti-Murhesa, Bideka and Burhale in Walungu. Their description is provided in Molima’s work [6].

### Study design and period

This is a cross-sectional study, using qualitative approach, conducted during two periods, from November 2017 to February 2018 and from November 2018 to February 2019 respectively (also coinciding with the monitoring periods of implementation of the project), with members of the community, in particular one of the members of the Health Area Development Committee (CODESA) concerned by the study (six members), as well as three adult patients met at the health care facilities of these six health areas.

### Study population and study criteria

Participants were selected for convenience. As this is a community-based study, we conducted it with members of the Health Area Development Committee (CODESA), which is one of the management bodies of the health center. This is a grouping of the Chairmen of the Community Animation Units (CAC) in the villages/towns that make up the health area. These CACs are made up of community health workers (CHW, Relais Communautaires or RECOs). The CODESA is the community participation body, representing the voice of the population. It is therefore responsible for ensuring contact between the health system and the community. It plays an important role in decision-making on the provision of care and the development of health services as well as in the co-management of health center resources with the health center team. The Community Health Worker is a member of the community, chosen in a village based on his dedication to the community, his morality, his availability, his personal motivation, his integrity, for the representation of the community in health activities [9, 24]*.*

Although CODESA is the community representative at the Health Centre level, we wanted to involve the patients, identified with the biopsychosocial problems, in the frame work of the PRD, the project on which this study is based, to also understand their perceptions, the way they perceive this care as direct beneficiaries of the intervention, in triangulation with the perceptions of the members of CODESA. At the time of the first data collection, patients with biopsychosocial problems were being identified, so they were not involved; but at the time of the second data collection, we wanted to hear from these patients who were in the health centers for care on the day of the interview, and who have agreed to participate in the study. The work carried out by Malembaka EB reproduces in its methodology the identification of these patients in the community [25].

### Theoretical framework

The Normalization Process Model (NPT) is a theoretical framework that aids in the understanding of how complex interventions are integrated and become feasible in everyday practice, particularly in health care [26–28].

We have used this model in this study as the biopsychosocial approach that is implemented aims at change in a complex system, taking into account the interactions between actors at different levels as well as their opinions throughout the process.

This model allows the analysis of the intended change process by describing the important factors that promote or hinder the implementation of complex interventions (interactional feasibility, relational integration, skill set feasibility and contextual integration) and by providing a basis for estimating the possibility of integrating a complex intervention into practice [29–32].

### Data collection tools and techniques

The use of the NoMAD tool adapted from NPT, annexed to this article (Annex 1), allowed us to collect data. With this tool, particular attention was paid to subjects dealing directly with the community, its relationship with the Health Centre, in relation to biopsychosocial care [29, 30].

We triangulated the data from the interviews with the members concerned to better analyze our study objective which was to know what they think about the understanding of the biopsychosocial approach by care providers through their daily practice over time and how they evaluate this approach in its implementation.

During the first period, the semi-structured interviews were conducted with members of the CODESA of the health areas concerned who represent the community at the health center. In the absence of a CODESA member (at Lumu and Burhale), the interview was conducted with a community health worker (RECO) active in community activities of the health center, in its health area, and was accessible. For the second period, in addition to the CODESA members, the interviews were also extended to patients present at the facilities on the day of the interview, who were direct beneficiaries of the intervention (specifically in Bideka, Kabushwa and Burhale). We conducted a total of 15 interviews, including 6 in the first period involving only CODESA members (November 2017-February 2018) and 9 in the second period, including 6 CODESAs and 3 patients (November 2018-February 2019). We used the same data collection tool (NoMAD tool) for both study periods, taking into account the collection of the same information from the same respondents (notably the members of CODESA), for their temporal comparison.

These interviews were conducted at the health center by the principal investigator, after obtaining informed consent signed by the interviewees (Annex 2). The interviewer took care to explain to each respondent the objective of the study in order to obtain his or her perception on the concept of holistic care (biopsychosocial approach) (BPS) at the health center. He then detailed the interview procedure, emphasizing the collection tool, the NoMAD, as well as the scoring of responses on a Likert scale ranging from 1 (not at all) to 5 (completely) and the reasoning to be provided for each question. Questions such as: "The management of biopsychosocial (BPS) situations requires a change in the way the health center works; The leaders of the health area are the driving force behind the management of BPS situations by the health center; I agree that the health center should manage BPS situations", formed the basis of the exchanges with the respondent, who was asked to give his or her point of view and to argue it. It should be noted that it was these explanations or responses that were more important to our study. When interviewing a patient, the interviewer focused more on questions concerning the care-giver relationship, and refrained from addressing other issues, deemed irrelevant to the patient. The different interviews lasted between 45 min and one hour, on average.

These interviews were conducted in French and Swahili by the interviewer. However, the respondent answered in the language that seemed easy to him or her, either in French or Swahili or in the local language (Shi). In the latter case, an interpreter facilitated the translation of the exchanges between the respondent and the interviewer. It should be noted that we used only one interpreter for all these interviews in the local language, from school of public health at the Catholic University of Bukavu, who speaks, understands and writes well. The interviewer took care to record the interviews using an audio recorder.

Regarding transcription, the interviews in French were transcribed directly into Word file by a research team member. Recorded interviews in Swahili and in the local language were transcribed as such before being translated by the interpreter. It should be noted that the transcriber participated in the analysis of these data.

### Data analysis

Our analysis was limited to the explanations that respondents provided based on the questions asked with the NoMAD tool (Annex 1); the scoring of responses was not analyzed. The data was analyzed manually. We carried out a thematic analysis using the NoMAD tool to build a thematic framework, based on the 16 questions in the NoMAD tool that respondents had answered. This framework included the following themes: (1) community impression on understanding and acceptance of the biopsychosocial approach by care providers, (2) role of community leaders in implementing the BPS approach, (3) mutual trust in implementing the BPS approach, (4) the BPS approach in the daily work of the Health Center (HC) agents and the community, (5) change in the way of working, (6) support for the approach for its implementation and sustainability, and evaluation of the BPS approach. Subsequently, these themes were grouped into categories based on their content, to facilitate the analysis. Thus, three categories were retained in the analysis: collective coordination, which groups together themes 1 and 2, leadership within the team, which groups together themes 3 and 4, and the integration of the biopsychosocial approach, which groups together themes 5 and 6.

Respondents were coded according to their health areas [A (CODESA) and A' (patient) for Bideka, B (CODESA) and B' (patient) for Burhale, C (CODESA) and C' (patient) for Kabushwa, D for CODESA Lumu, E for CODESA Lwiro and F for CODESA Nyamuhinga], the survey period (1 for the first period, from November 2017 to February 2018, and 2 for the second period, from November 2018 to February 2019) and the number corresponding to the respondent's answer (1 to 16) based on the NoMAD tool adapted to facilitate data analysis. Thus, the verbatim from the interviews that were used for the analyses were systematically numbered according to the three parameters listed. For a Lumu respondent interviewed in the first period whose response corresponded to the fourth question of the NoMAD tool was coded as 'D14', and for the same respondent in the second period for the fourteenth question of the NoMAD tool, the code was 'D214'.

## Results

The following table (Table 1) gives a description of the general characteristics of our sample.

**Table 1:** General characteristics of respondents

Variable	Number	
	CODESA	Patient
**Gender**		
Male	3	1
Female	3	2
**Age**		
Under 50 years	2	
Between 50 et 65 years	4	3
**Civil status**		
unmarried	1	
Married	5	2
Widow		1
**Educational level**		
Primary		1
Secondary	5	2
Superior/University	1	
**Occupation**		
Unmployed	1	
Merchant	1	1
Farmer	2	2
Official/Teacher	2	

### Collective coordination

#### Community impression on understanding and acceptance of the biopsychosocial approach by care providers

According to the community responses, their perception was that the providers’ understanding of the biopsychosocial approach was not the same at the beginning of the implementation.

From the interaction with the health workers, some community members found that the approach was clear t providers. The latter already knew approximately what each of them had to do to manage patients with complex problems.

"…they come in large numbers, people with blood pressure come, people with diabetes come… people with tuberculosis, people with AIDS, pregnant women come… I can see that they (the providers) know, for them it's clear… if they see a patient who is suffering a lot, we put him before the others to save his situation…" D13.

Although providers understand this approach and know what they are being asked to do, capacity building in some areas is needed to ensure better holistic care for patients with complex situations.

"They may know what to do… they have an idea but need capacity building on the psychological side. The moment the psychologist is not there, if the nurses are trained they can do it…" C12.

For others, however, the impression at the beginning was that the health staff did not yet have a clear idea about the biopsychosocial approach. It was not clear to the providers what each should do.

"I don't think the handling of biopsychosocial situations is clear. For the workers who work at the health center as I know them, as I am used to interacting with them, I think their capacity needs retraining to do so." F13.

Subsequently, with the implementation, this approach seemed to be well understood by the providers, based on the testimonies of the patients who regularly attend the health facility for care *"… this approach is well mastered by the nurses because they take care of us properly and explain things very well and that helps us to get good care…". B'*23 .

Although understood, this retraining or reinforcement of skills was necessary for all providers and not just a few team members, in order to allow a common understanding of the approach by all .

"… only some of them had been trained, but they all had to be trained for the care to work well. And if the patient managed to meet the one who had not been trained? F23 "… it could be good if the whole team knows how to manage these patients in relation to this care…". E23.

On the basis of the information obtained during both the first and second periods, the community has shown acceptance of the biopsychosocial approach to care and its integration, as it also benefits from it, taking into account its role and responsibility towards the patient and the care structure. Once the patient is referred from the community to the health center, he is taken care of overall, and if necessary, he is referred for continuity of care, in case his problem goes beyond the minimum package of care.

"… and we as representatives of the community accept; it can also help us because we are the ones who bring the patients to the health center when we have already sensitized them in the community, and it is the health center that refers them to the hospital in case they are unable…". A18.

#### Place of community leaders in the implementation of the BPS approach

The community has a great deal of influence in the implementation of activities at the health center, through the community participation bodies. All the respondents stated, during the two periods concerned, that the leaders are the driving force behind the management of BPS situations by the health center. Certain fundamental elements were mentioned, notably the representation of the community at the HC level, which requires good collaboration and involvement *"…they (the leaders) are the community and the HC takes care of the community. They collaborate with the HC…" C15 "…it is through them that the community is sensitized and this contributes to the smooth running of the approach…" B25*; the role that leaders play in sensitizing the community to adhere to and support the activities of the health center *"…in our community our job is to direct people to the facilities and those in the facilities (providers) cannot be everywhere at once and they are very overloaded, so it is up to the community leaders to sensitize their people… E110.*

"The leader must first bring the new practice of the health center to the community and in addition he must refer the patient to the health center by reassuring him of the quality of care that will be provided at the health center. F15.

And as they expressed it, the activities related to the BPS approach are integrated in the community. Leaders are already engaged in activities such as referring patients with complex problems, community outreach, home visits for patients… *"…as a representative of my community, I mobilize the community relays to be active, to ensure referral of patients and home visits of patients…" A110 "…I do my job, I make home visits and we talk about it with the health center…" F210.*

### Leadership in the HC team

#### Mutual trust and burden sharing in the implementation of the biopsychosocial approach

Responsibilities within the health team are shared according to each person's area of expertise, and this builds confidence in the work; this is supported by the community. Community representatives can challenge health workers if they observe behavioral gaps in care: *"And if we think there’s something wrong, we soon tell the IT that we’ve noticed this kind of behavior with nurse. Or we hear from the sick we sent to the health center, he will tell you I was not well received in your health center… Or the way I was received I was not satisfied… you ask him which nurse received you, he will say this is so, he is so tall, because we know our nurses. " D111.*

Although responsibilities are shared, the community wants versatility within the care team, in the sense that they can replace each other if they are unable to work without compromising the implementation of activities. *"I would like everyone to be trained; if we train only one person, we may have a problem if he is ill or transferred… it is important that there is a distribution but that everyone is capable of doing it" F112.*

The community believes that the lack of collaboration between the IT and the other agents has an impact on trust, and this can act as a brake on the implementation of the approach, as there will not be fluid communication within the team: *"The IT had to collaborate a lot with the HC agents, the staff; because you cannot work without being informed; otherwise you can work vaguely. The officers know, it's almost true, the officers are informed but they may think that since they are not involved in this work they are not involved at all." F112.*

#### Biopsychosocial approach in the daily work of the Health Center workers and the community

For some community representatives, although they believe that dealing with biopsychosocial situations is part of the daily work of health workers, the latter should only focus on the biopsychological side, and provide support to the community for the social aspect. Collaboration and complementarity between health workers and the community are encouraged to make this approach effective: *"… when we educate patients to go to the health center, the IT gives the medication and advice… We go to them at home, the ones with diabetes…they tell us if sugar has gone up or not, and we inform the IT…" B17.*

On the other hand, for others, providers will have to internalize this approach and integrate it into their daily activities, because some patients who come to the health center, their problems do not necessarily require medication, but it is more psychological and social: *"…a mother came to the center with stomach aches, headaches … and after the examinations … all the results were negative. And in the end we realized that the mother is a widow and that her son was trying to sell his house… The Head Nurse called her son, … and after learning that her son is not going to sell the house anymore, all the selling and headaches disappeared…" D17.*

One of the fears regarding the integration of the approach into the daily activities of the health center is that it will be considered as an additional job, which in this case requires an additional premium for its implementation, as expressed by this respondent, arguing for the providers: *"…because when there is added activity, we have to think about what that means, the more we work, the more we are paid… And then, as the activity increases, so does their premium" A17.*

### Integration of the BPS approach

#### Change in the way of working at the health center

The community believes that the management of biopsychosocial situations requires a change in the way health workers work, from the reception of the patient to their discharge from the facility. "*…there are certain agents who do not welcome patients well… the welcome was not warm, so the patient is not at all satisfied, so I would say that we need to improve the way we take care of patients at the health center… C11.*

The care of patients at the health center was more focused on the somatic level, with providers only prescribing medication on the basis of the clinic and laboratory results. In addition to the medical problems, the patient needs other services for his well-being, already feeling left out himself… He thinks he will find the remedy with the health professional he confides in, which requires consideration from the health workers. *"Some identified patients, when you see them on their face, you can read messages of discontent, messages that express … anxiety, … the patient … stigmatizes himself, it's already a problem… I don't see a single patient of this type… who arrives at the health center without needing this psycho-social care" D11.*

This change in the way of working encourages the community to see added value in the work of the providers through the implementation of the biopsychosocial approach. In addition to capacity building, facing these complex situations and seeking a solution further strengthens their performance, and this is appreciated by the community. "*What is being done is awakening their consciousness and everywhere they go if they see another patient they are interested and it is like a step that is added in our activities… and it strengthens their capacity…". A14.*

So, over time, this change is noticed even by the patients who encourage this new approach to care *"… and during the consultations they also address the psychological issues and therefore go in depth to check the background of the problem and they explain everything to us in detail…" A'21.*

"I would be really happy if it was applied for other patients and not only for diabetics and hypotensive and even for people who suffer from gastritis and for all diseases it can help them.

#### Support for the BPS approach in its implementation and sustainability, and evaluation of the approach

The various stakeholders are called upon to support the BPS approach in its implementation. 

The Health District authorities (the District Management Team), as the drivers of the approach, are called upon to be involved to support and sustain it. The monthly primary health care meetings (monthly review) as well as the supervisions organized by the members of the District Health Management Team are the occasions where the approach can be discussed for improvement and support. "*… in the monthly reviews at the health district level, cases are raised that require further follow-up in relation to the provision of the services we have in the facility, the IT can identify a case and say so in the review meeting…" E16.*

In this same monitoring framework, support from the provincial health authorities is essential. This support is noted by some community leaders *"…since the provincial office comes here to supervise, to look at the situations of all these care-givers…". F113 "… the Provincial Health Division accompanies the program with the supervision of the agents, it is informed of this approach…". C213.*

On the other hand, for others, this support from the provincial level is difficult to appreciate, especially because of the lack of certain information, due to the fact that the community is not permanently at the Health Center *"… I don't know if it's because I'm not here permanently, but I'm not informed by the IT if the provincial office has already arrived here for this care…". D113.*

Community leaders expressed their desire to see the approach become sustainable, while integrating community activities, in support of the approach, provided that certain resources are made available *"… if we are not supported with a small amount of money we cannot succeed…". F113*, such as the development of certain mechanisms to ensure financial autonomy and mutual support *"… but we can also set up an AVEC (village savings and credit association) so that the money we have can help in this activity of caring for these patients… we need to be trained in how to finance ourselves. A19.*

As beneficiaries of the interventions, the community is best placed to provide feedback on the effects of the interventions. They are the users of the services, and as such, they are the primary evaluators of the services provided by the providers. The community representatives, who are in contact with the health authorities, collect information from the service users on the quality of the interventions offered to them, which they pass on to the providers *"…because the way we work here, we bring in patients and then we ask the patients how they were received and treated and then we go and tell the nurse not to do this anymore or to improve this…". C111 "As a community leader, when a patient comes from the health center, I ask him how he was received or treated and from there I have my assessment…". E114* .

For some representatives, the implementation of the approach is obvious, and this is even noticeable in the change in the work of the health center team following the evaluation of the approach. The improvement of the working environment through support for materials and equipment is also mentioned as an element of support for the approach *"… before they didn't have these test materials, … they were shown how to use these devices, especially the scales there and they saw their importance and that's how this modification became apparent…". A116.*

For others, on the other hand, it is early to be able to evaluate the effects of the biopsychosocial approach and to talk about changes in the team's work as a result of the evaluation, especially since this is the beginning of the implementation. "*… I don't know yet, because it's the beginning, but it's important that they can adapt their way of working…" D116.*

But over time, as the data from the second period show, the community noticed a change in the way the health care team worked. They had adapted to the organization of their work in order to facilitate consultations and treatment according to the new approach. "… *the patients tell us that when they go to the health center, the nurses want to know their health problem in depth. They also deal with their psychosocial problems… We hope that this approach will continue because it is good for the community…" D 29.*

Others even noted that providers improved communication among themselves. They exchanged their experiences with the implementation of the approach and discussed different complex cases in order to improve the management of their patients. "… *the agents of the center help each other, they discuss with each other, they give each other advice to work well…" E216.*

These elements support the implementation of the BPS approach.

## Discussion

The results of our study show us the importance of the community in implementation of the biopsychosocial approach to health care and its integration at the level of the health center. They also provide us with the elements mentioned by the community, either in the first period of the study or in the second period, some of which may constitute a barrier to implementation (lack of understanding of the approach by providers, lack of collaboration between providers on the one hand, and between providers and the community on the other hand, poor reception of patients, lack of involvement of the community, lack of communication between providers and community, overload or additional work among providers requiring an additional premium…), and others facilitate it (strengthening of skills, complementarity in work, collaboration and fluid communication between the community and providers, support at the higher level, support for financial autonomy and mutual support…). These elements should be taken into account for both providers and the community in improving the quality of care and accessibility to quality care services [33, 34].

### The role of the community and its involvement in the implementation of BPS approach and its sustainability

The community plays a key role in the implementation of facility-based interventions. As the results of our study show, she has a say, she gives her impression of what the health facility offers her. This perception may lead them either to adhere to these interventions and be able to support them, or, if not, itself constitute a brake on the success of the intervention. In addition to this, she has a view of the way in which the care is provided, one of the elements for assessing the effectiveness of the care, as mentioned by certain authors [35–37].

We saw among our respondents that the leaders were identified among the driving forces for the management of biopsychosocial situations by the health center. It was mentioned that good collaboration between providers and community actors increases the level of community dynamism in health activities [38]. Experience has shown that the involvement of beneficiaries in the provision of care through a social responsibility mechanism that involves users in improving the provision of care can be a lever for improving access to and quality of care [39].

In order to support the approach and ensure its integration, community representatives have shown their commitment to take ownership of it and ensure its sustainability. The community activities they carry out (patient orientation, awareness raising on health promotion and other activities of the health center, home visits…) already testify to this, and the fact that they themselves use the services offered there, setting a good example for all as mentioned by Allen A.J et al. [40].

Other experiences indicate the involvement of the community in solving health problems, supporting the health system in complex situations where resources are limited [41]. Community involvement is therefore essential for the implementation of an intervention in the health sector, and its real integration, as some studies and innovative experiences also show us [42, 43].

The community leaders express their wish to see the approach become permanent within the community, provided that certain means are made available or certain mechanisms are developed to ensure financial autonomy and mutual support, such as the Village Savings and Credit Associations (AVEC). This is a form of community solidarity of microfinance to meet certain basic family needs (paying for food, schooling, care, etc.) through financial assistance [44–46].

This form of mutual support leads to good results, especially for patients in complex situations, requiring holistic/biopsychosocial care [47].

### Patient flow, provider versatility, multidisciplinary and capacity building

The community recognizes that the health center is the patient's entry point into the health system, and therefore the starting point for all care, including biopsychosocial care. First aid is provided at the health center, before referral for continuity of care, especially for cases that fall outside the minimum care package. In this context, a good doctor-patient relationship is encouraged, in the sense of adherence to the treatment, taking into account the patient's concerns, and this will allow for successful treatment. 

Manzambi et al. showed in their study on the determinants of health center use in urban Africa that patients use the health center more than elsewhere when they are looking for, among other things, versatility in treatment [48].

An action-research study conducted among TB patients in 6 French-speaking African countries has shown that collaboration between health care providers and patients leads to good results in treatment. Faced with various situations (non-adherence to treatment, non-compliance with the referral, abandonment of treatment, etc.), a series of strategies is proposed to respond to the problems identified (reorganization of the patient circuit, taking account of patients' concerns, home visits, etc.) and constitutes success in the management [49].

Although the approach is integrated, the need for capacity building was mentioned by most of our respondents. In the provision of quality care, continuing education is an indispensable asset for good patient care. For example, the WHO advocates for the integration of certain essential services into primary health care, capacity building of health care providers to ensure quality care, and delegation and task sharing [50].

A survey of mental health care providers in South Africa identified priority areas for strengthening their skills to improve mental health care [51].

This capacity building should not be limited only to providers, but also to community relays who are representatives of the community, and who have an important role in the implementation of the biopsychosocial approach. Attinsounon, in his study conducted among community relays on Lassa and Ebola hemorrhagic fevers in Benin, shows that their capacity building could contribute not only to improve their knowledge on these deadly epidemics but especially to improve the quality of their interventions in the community [52].

One of the fears expressed by the community about the approach and its integration into the daily activities of the care facility is that the provider sees it as an extra job, which requires an extra premium for its implementation. The community also felt that the lack of collaboration between the IT and other staff had an impact on trust, and this could be a hindrance to the implementation of the approach, due to the lack of smooth communication within the team. These elements were also mentioned by Molima et al. [6].

### Evaluation/assessment of health workers' work (the performance)

As we have seen with our results, the community is best placed to give its impression of the effects of the interventions, as the direct beneficiaries of the interventions. They are the primary evaluators of the services provided by the providers. Community evaluation of health workers is commonly practiced in the context of performance-based financing (PBF), with service beneficiary satisfaction surveys or community auditing. Various studies have shown the importance of community auditing in improving the provision of quality care and even make suggestions for its implementation, especially in order to avoid its drawbacks [53, 54].

However, this community assessment is limited to certain aspects of the intervention, as the community, being outside the field, will not be able to address the main elements of quality of care, due to lack of certain information. Kumbani also shows us the limitations of community assessment of quality of care in his study in Malawi [55].

Another form of performance evaluation is supervision by the hierarchy. As we have seen from our results, the district management team, as the drivers of the approach, is called upon to support and sustain it. The monthly reviews as well as the supervisions organized by the members of the health district management team are the occasions during which the approach can be evaluated for improvement, integrating also the community aspects. In this same monitoring framework, support from both national and provincial health authorities is essential. Some authors have explored the support and supervision approach in this evaluation framework, have even proposed tools for its application, and have analyzed the impact of these supervisions in relation to the quality of the care offered [56–58].

### Limitations of the study and operational implications

We were not able to compare the statements of the respondents in relation to practice (awareness raising, home visits, etc.). It should be noted that the understanding of the approach, based on our collected data, is that of the care providers, as we are told by the respondents. They explain how they perceive providers’ understanding of the BPS approach, based on certain aspects of care (team work, quality of work, change in the way of doing things, mutual trust…) without going into in depth the elements of improving the quality of care and community participation, as proposed by Kelley and Hurst [59]. In addition to the method used with the NoMAD tool, we could have broadened our study to ensure real community involvement by also integrating "design thinking", an emerging instrument that is being promoted by researchers to analyze community involvement in the implementation of health interventions [43]. The use of a single interpreter could limit the understanding or accurate translation of our respondents' statements. With our sample size, not all the necessary information may have been analyzed for this study. It will need to be expanded in a subsequent study.

The results of our study should encourage the involvement of community participation in any process of integrating the biopsychosocial model of health care. All other things being equal, other levels of the health system should apply this BPS approach.

## Conclusion

This survey of community representatives and some patients on the biopsychosocial approach enabled us to understand the community's perceptions on this approach and its integration at the health center. With our results, we have also identified both professional and community elements that act as a barrier to the implementation of the biopsychosocial approach, as well as others that favor its implementation, and these elements should be taken into account, in the process of integrating the biopsychosocial model of person-centered health care with a view to improving the quality of care and community participation. Some practices are encouraged to support the approach, including the development of some mechanisms to ensure financial autonomy and mutual support, notably the AVEC. As we have seen, the study did not sufficiently address these elements of improving the quality of care and accessibility to quality care services. Further in-depth studies will measure community engagement and explore the two aspects mentioned above in implementing the biopsychosocial approach.

### Supplementary Information

**Additional file 1: Annex 1.** NoMAD tool focused on the management of Biopsychosocial situations (inspired on Normalization Process Theory).

**Additional file 2: Annex 2. **Research project: Organizational analysis of the management of complex psycho-medicosocial situations in South Kivu.

## Data Availability

The datasets used and/or analyzed during the current study are available from the corresponding author on reasonable request.

## Abbreviations

AVEC: Village savings and credit association
BPC: Biopsychosocial
CAC: Community Animation Units
CHW (RECO): Community health workers (Relais Communautaires)
CODESA: Health Area Development Committee
DRC: Democratic Republic of Congo
HA: Health areas
HC: Health centers
HD: Health Districts
NoMAD: Normalization MeAsure Development
PBF: Performance based financing
