# Supplementary material for: Community perceptions of a biopsychosocial model of integrated care in the health center: the case of 4 health districts in South Kivu, Democratic Republic of Congo
Source: BMC Health Serv Res. 2023 Dec 18;23:1431. doi: 10.1186/s12913-023-10455-1 (PMC10726631; doi:10.1186/s12913-023-10455-1)
Supplement: Supplementary file 1 — Additional file 1: Annex 1. NoMAD tool focused on the management of Biopsychosocial situations (inspired on Normalization Process Theory). [file 12913_2023_10455_MOESM1_ESM.docx]

NoMAD tool focused on the management of Biopsychosocial situations (inspired on Normalization Process Theory)

1/ The management of biopsychosocial situations requires a change in the way i work.

| Not at all |  |  |  | Absolutely |
| --- | --- | --- | --- | --- |
| 1 | 2 | 3 | 4 | 5 |

Explain

2/ Health center wokers agree on the goal of taking an interest in the care of biopsychosocial situations at the health center level

| Not at all |  |  |  | Absolutely |
| --- | --- | --- | --- | --- |
| 1 | 2 | 3 | 4 | 5 |

Explain

3/ What the health center’s care of biopsychosocial situations requires of me is clear

| Not at all |  |  |  | Absolutely |
| --- | --- | --- | --- | --- |
| 1 | 2 | 3 | 4 | 5 |

Explain

4/ The care of biopsychosocial situations by the health center brings a clear added value to my work

| Not at all |  |  |  | Absolutely |
| --- | --- | --- | --- | --- |
| 1 | 2 | 3 | 4 | 5 |

Explain

5/ Community leaders in the Health area are the driving force behind health center’s care of biopsychosocial situations.

| Not at all |  |  |  | Absolutely |
| --- | --- | --- | --- | --- |
| 1 | 2 | 3 | 4 | 5 |

Explain

6/ The authorities of the health center and the health district are driving forces for the care of biopsychosocial situations by the health center

| Not at all |  |  |  | Absolutely |
| --- | --- | --- | --- | --- |
| 1 | 2 | 3 | 4 | 5 |

Explain

7/ I agree that the health center’s handling of biopsychosocial situations is part of my work

| Not at all |  |  |  | Absolutely |
| --- | --- | --- | --- | --- |
| 1 | 2 | 3 | 4 | 5 |

Explain

8/ I agree that the health center should deal with biopsychosocial situations

| Not at all |  |  |  | Absolutely |
| --- | --- | --- | --- | --- |
| 1 | 2 | 3 | 4 | 5 |

Explain

9/ I will continue to support this care even after some time that the health center has started to take care of biopsychosocial situations

| Not at all |  |  |  | Absolutely |
| --- | --- | --- | --- | --- |
| 1 | 2 | 3 | 4 | 5 |

Explain

10/ I do the necessary activities to manage biopsychosocial situations

| Not at all |  |  |  | Absolutely |
| --- | --- | --- | --- | --- |
| 1 | 2 | 3 | 4 | 5 |

Explain

11/ I am confident in the work and skills of the other health center members in dealing with biopsychosocial situations

| Not at all |  |  |  | Absolutely |
| --- | --- | --- | --- | --- |
| 1 | 2 | 3 | 4 | 5 |

Explain

12/ The activities for the care of biopsychosocial situations by the health center are distributed equally among the various members of the health center

| Not at all |  |  |  | Absolutely |
| --- | --- | --- | --- | --- |
| 1 | 2 | 3 | 4 | 5 |

Explain

13/ The health center is adequately supported by the authorities to organize the care of biopsychosocial situations

| Not at all |  |  |  | Absolutely |
| --- | --- | --- | --- | --- |
| 1 | 2 | 3 | 4 | 5 |

Explain

14/ I have the necessary information to evaluate the effects of the management of biopsychosocial situations by the health center

| Not at all |  |  |  | Absolutely |
| --- | --- | --- | --- | --- |
| 1 | 2 | 3 | 4 | 5 |

Explain

15/ The team at the health center believes that it is worthwhile for the health center to take care of biopsychosocial situations

| Not at all |  |  |  | Absolutely |
| --- | --- | --- | --- | --- |
| 1 | 2 | 3 | 4 | 5 |

Explain

16/ The team at the health center changes their work after evaluating the management of biopsychosocial situations

| Not at all |  |  |  | Absolutely |
| --- | --- | --- | --- | --- |
| 1 | 2 | 3 | 4 | 5 |

Explain
