# Supplementary material for: Community perceptions of a biopsychosocial model of integrated care in the health center: the case of 4 health districts in South Kivu, Democratic Republic of Congo
Source: BMC Health Serv Res. 2023 Dec 18;23:1431. doi: 10.1186/s12913-023-10455-1 (PMC10726631; doi:10.1186/s12913-023-10455-1)
Supplement: Supplementary file 2 — Additional file 2: Annex 2. Research project: Organizational analysis of the management of complex psycho-medicosocial situations in South Kivu. [file 12913_2023_10455_MOESM2_ESM.docx]

*Research project: Organizational analysis of the management of complex psycho-medicosocial situations in South Kivu*

*Informed consent form*

**1. Title of the study**: Organizational analysis of the management of complex psycho-medicosocial situations at the front line of care in South Kivu: the case of chronic illnesses and the malnourished mother-child pair.

**2. Research team**

The study is being conducted as part of a Development Research Project bringing together researchers from three universities (UCB, UCL and ULB) and a Congolese research center (CRSN-Lwiro).

Principal investigator : Christian MOLIMA EBOMA - Researcher at the School of Public Health of the Catholic University of Bukavu and doctoral student.

**3. Introduction**

We invite you to take part in a research study. It will take place in six rural health areas in South Kivu (Bideka, Burhale, Kabushwa, Lumu, Lwiro, Nyamuhinga).

Before you decide to take part, we'd like to explain what it will be used for and how it will be implemented. Your participation in our research is entirely voluntary; you can choose to take part or not. Either way, it will not affect support for the health system and primary care structures.

You can take your time before confirming your participation. You can discuss it with other people (colleagues, friends, family, medical staff, etc.). If you have any questions or concerns about taking part in our research, please let us know.

**4. Aim of the study**

The aim of this study is to gain a better understanding of the various factors that make it difficult to provide appropriate care for medical and psychosocial situations in this health area, and to identify changes that would improve care, for example for mothers of malnourished children or patients with diabetes and/or high blood pressure with psychosocial problems. The research is interested in your knowledge of the current organization of care, the difficulties encountered in the management of multi-morbidities and malnutrition, and your expectations for the future and in the future to provide better care.

This study will provide information for improving the organization of medical and psychosocial care services (treatment, social integration and improving quality of life) for future patients in this situation.

**5. Explanation of the survey procedure**

For this research, we are going to ask you a series of questions:

a. About your role in the care system

b. On the current organization of care in the health centers

c. On the current challenges facing the province's primary care structures

d. On the support to be given to the care system to ensure comprehensive care for people in complex psycho-medico-social situations.

We will be discussing these issues with you today, and 1 or 2 more times if we feel that you could add to them. The aim is always to gather as much information as possible on the current organization of care and on the dynamics of possible changes.

The whole process will take around 45 minutes to 1 hour. The questions we ask you are fairly simple; there are no right or wrong answers: only your point of view counts.

Please note that the entire interview will be recorded on a tape recorder so that we can transcribe the entire conversation faithfully.

We're going to give you some sentences, you're going to give us your opinion and then your position on certain hypotheses, and then we'll ask you why you think this sentence is correct, or not correct at all, or only half correct. These explanations are important for our research.

**6. Participant's rights**

Participation in this research is completely free and voluntary. Even if you decide to take part, you are not obliged to answer any questions that make you uncomfortable. If this is the case, tell us that you do not wish to answer this question and we will move on to the next question. You can stop taking part in our research at any time: just contact us and confirm your decision.

At the end of each meeting, we could listen to the recording again together if you agree. At this stage, you are free to decide whether to continue the research or to stop it. In this case, we would just like to know the reason why.

**7. Confidentiality**

The information we collect in the course of this research will be faithfully transcribed and stored in a computer file. Only the research team will have access to these files. The files will be secure (kept under lock and key or with a password).

Your name will not appear on any document (apart from the identification form). Your name will be replaced by a code that guarantees your anonymity. When we analyze the information, this code will always appear - never your name.

All the information you give us is strictly confidential. No one will know what you have told us (neither medical staff, nor health or administrative authorities, nor partners supporting health zones).

**8. Problems or questions**

If you have any problems or questions about this research, you can contact the principal investigator. You can contact us either during our visits to the center; or by telephone.

Christian Molima : +243997717425 ; Email : molimachris@gmail.com

Problems or questions concerning this research can also be addressed to the Ethics Committee of the Catholic University of Bukavu.

Prof. Eloi Macece - President of the Ethics Committee, Tel : +243992838793 ; Email : macece.bagendabanga@ucbukavu.ac.cd

**9. Informed consent**

I have heard and understood the above information. All my questions regarding this research have been answered. I agree to take part in/renew my consent to take part in the research project "Organizational analysis of the management of complex psycho-medicosocial situations in South Kivu".

Last name and first name of participant Date:

Signature or fingerprint

In the case of a non-emancipated minor participant :

Last name and first name of an adult referee

Signature or fingerprint Date

**10. Principal investigator's undertaking and signature**

I certify that I have explained to the participant the terms of this information and consent form, that I have answered any questions the participant may have in this regard and that I have made it clear to the participant that he/she remains free to end his/her participation without any negative consequences. I undertake with the research team to respect what has been agreed in the information and consent form and to give a signed copy to the participant.`

_____________________________________________________

Name of researcher responsible for the research project Date :

_____________________________________________________

Signature of the researcher responsible for the research project
